# Dataset of genome sequencing and amoxicillin degradation data of *Bacillus cereus* BT-C2.4 isolated from aquaculture water

**DOI:** 10.1016/j.dib.2026.113083

**Published:** 2026-07-15

**Authors:** Hong Lat Luu, Nhu Nguyet Phan, Thanh Nhan Lam, Trang Thi Phuong Phan

**Affiliations:** a Center for Bioscience and Biotechnology, University of Science, Ho Chi Minh City, Vietnam; b Vietnam National University, Ho Chi Minh City, Vietnam; c National Hospital of Odonto - Stomatology, Ho Chi Minh City, Vietnam; d Institute of Public Health, Ho Chi Minh City, Vietnam

**Keywords:** Antimicrobial resistance, Environmental microbiology, Β-lactam resistance, Aquaculture systems, Antibiotic biodegradation

## Abstract

This dataset describes the draft genome sequence and experimental amoxicillin degradation data of *B. cereus* BT-C2.4, isolated from aquaculture water in Ben Tre province, Vietnam. The genome was sequenced using Illumina technology and assembled into 61 contigs with a total length of approximately 5.44 Mb and GC content of 35%. Genome annotation was performed using the NCBI Prokaryotic Genome Annotation Pipeline (PGAP). The dataset also includes high performance liquid chromatography (HPLC) data measuring residual amoxicillin concentrations over time, showing that amoxicillin was fully degraded within 24 h under the tested conditions. These data may be useful for studies on antimicrobial resistance, antibiotic biodegradation, and environmental microbiology in aquaculture systems.

Specifications Table

**Table:** 

Subject	Biology
Specific subject area	Microbial genomics and antimicrobial resistance in aquaculture environments
Type of data	Genome sequences; tables; figures; HPLC data (raw and analyzed)
Data collection	*B. cereus* BT-C2.4 was isolated from aquaculture water samples. Whole genome sequencing was performed using the Illumina MiniSeq platform. Amoxicillin degradation was evaluated using HPLC analysis under controlled laboratory conditions.
Data source location	Institution: University of Science, Vietnam National University, Ho Chi Minh City City: Ho Chi Minh City Country: Vietnam Sample collection site: Ben Tre Province, Vietnam
Data accessibility	Repository name: GenBank, NCBI Sequence Read Archive (SRA), and Mendeley Data Data identification number: GenBank accession JBMDTX000000000; BioProject: PRJNA1230794; Mendeley Data DOI: 10.17632/5zs2c96bp4.2 Direct URL to data: https://www.ncbi.nlm.nih.gov/bioproject/PRJNA1230794; https://doi.org/10.17632/5zs2c96bp4.2 Instructions for accessing these data: All data are publicly available without restriction.
Related research article	None

## 1 Value of the Data

• Provides genomic and experimental data that may support future studies of antimicrobial resistance and antibiotic degradation in environmental Bacillus isolates.
• Offers a reference genome for *B. cereus* strains isolated from aquaculture environments in Vietnam
• Enables comparative analysis of antimicrobial resistance and biodegradation mechanisms across environmental isolates
• Supports future research on antibiotic removal and bioremediation strategies in aquatic systems

## 2 Background

The extensive use of antibiotics in aquaculture has contributed to the accumulation of antibiotic residues and the emergence of antimicrobial resistance in aquatic environments. Amoxicillin is one of the commonly used antibiotics, and its persistence in water systems poses environmental and public health concerns. Microorganisms capable of degrading antibiotics may play an important role in mitigating these effects. *B. cereus* is widely distributed in environmental habitats and is known to possess intrinsic antimicrobial resistance mechanisms. However, datasets linking genomic characteristics with antibiotic degradation capacity remain limited, particularly for strains isolated from aquaculture environments in Vietnam.

This dataset was generated to provide both genomic and experimental data describing the amoxicillin degradation capability of *B. cereus* BT-C2.4, enabling further studies on antimicrobial resistance and environmental bioremediation.

## 3 Data Description

**Fig. 1:**
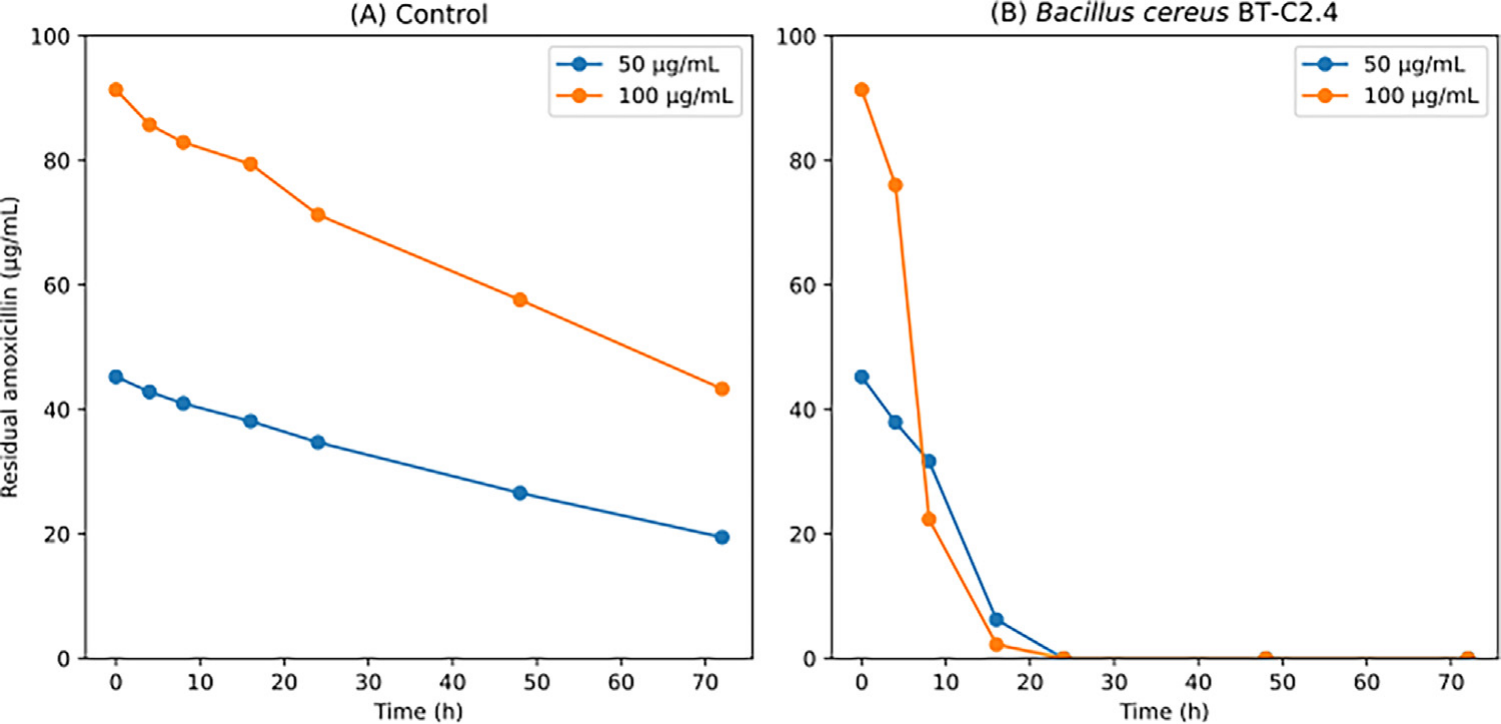
Residual concentration of amoxicillin during degradation by *B. cereus* BT-C2.4 measured by HPLC. Amoxicillin was prepared at nominal concentrations of 50 and 100 µg/mL; the measured concentrations at 0 h were 45.25 and 91.36 µg/mL, respectively. Samples were collected at defined time intervals. Complete degradation was observed within 24 h under the tested conditions.

The dataset includes experimental and genomic data for B. cereus BT-C2.4. Amoxicillin degradation experiments were performed using a shake-flask system. Briefly, 50 mL of sterilized shrimp pond water was transferred into 250-mL Erlenmeyer flasks and supplemented with amoxicillin at nominal concentrations of 50 and 100 µg/mL. Amoxicillin stock solutions were prepared from amoxicillin trihydrate dissolved in sterile distilled water and subsequently diluted to the desired concentrations before inoculation. The isolate was cultured on tryptic soy agar containing amoxicillin (50 µg/mL), and bacterial suspensions were adjusted to approximately 10⁸ CFU/mL (0.5 McFarland standard). Each flask was inoculated with 1 mL of the bacterial suspension and incubated at 30 °C with shaking at 120 rpm. Uninoculated flasks containing the same amoxicillin concentrations served as controls. The amoxicillin degradation profile is shown in Fig. 1. Degradation experiments were performed in sterilized shrimp pond water containing amoxicillin at nominal concentrations of 50 and 100 µg/mL. Cultures (∼10^8^ CFU/mL) were incubated at 30 °C with shaking, with uninoculated controls included. Samples were collected at defined time intervals over (0 – 72 h), filtered (0.45 µm), and analyzed by HPLC using a C18 column with UV detection at 229 nm and a mobile phase of acetonitrile:water (30:70, v/v, 0.1% HCl). Concentrations were calculated from peak areas using an external calibration curve, and measured 0 h values were used as baseline. Minor differences between nominal and measured concentrations likely reflected routine experimental variation.

A previous study by Duong-Nguyen et al. (2022) reported substantial amoxicillin degradation by a *Bacillus cereus* strain isolated from aquaculture sludge under optimized laboratory conditions. Consistent with that observation, *B. cereus* BT-C2.4 also demonstrated efficient amoxicillin removal in the present study. In addition to degradation data, the current dataset provides whole-genome sequence information, genome annotation, ANI analyses, antimicrobial resistance genes, virulence-associated genes, and predicted biosynthetic gene clusters, thereby expanding the genomic resources available for environmental Bacillus isolates associated with antibiotic degradation.

The genome assembly dataset includes a total genome size of 5441,081 bp, assembled into 61 contigs with a GC content of 34.99%. Genome assembly statistics are summarized in Table 1.

**Table 1:** Genome assembly statistics of *B. cereus* BT-C2.4.

Feature	Value
Genome size	5441,081 bp
Contigs	61
Largest contig	849,517 bp
GC content	34.99%
N50	341,642 bp
CDS	5367
tRNA	86
rRNA	8
Coverage	85x
Total genes	5610

**Fig. 2:**
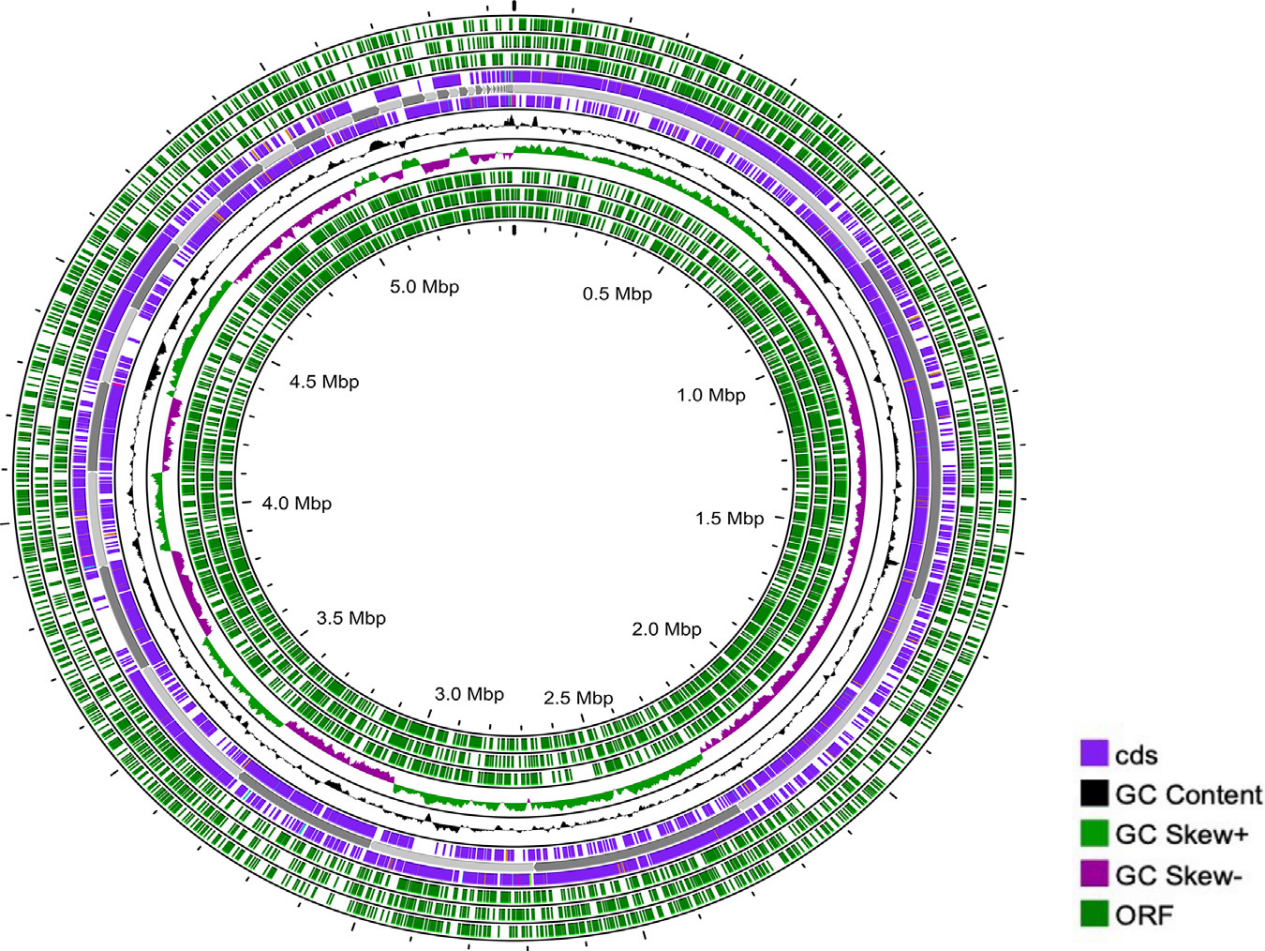
Circular genome map of *B. cereus* BT-C2.4 showing coding sequences, GC content, and genomic features. The map was generated using Proksee.

A circular genome map illustrating coding sequences, GC content, and GC skew is shown in Fig. 2

**Fig. 3:**
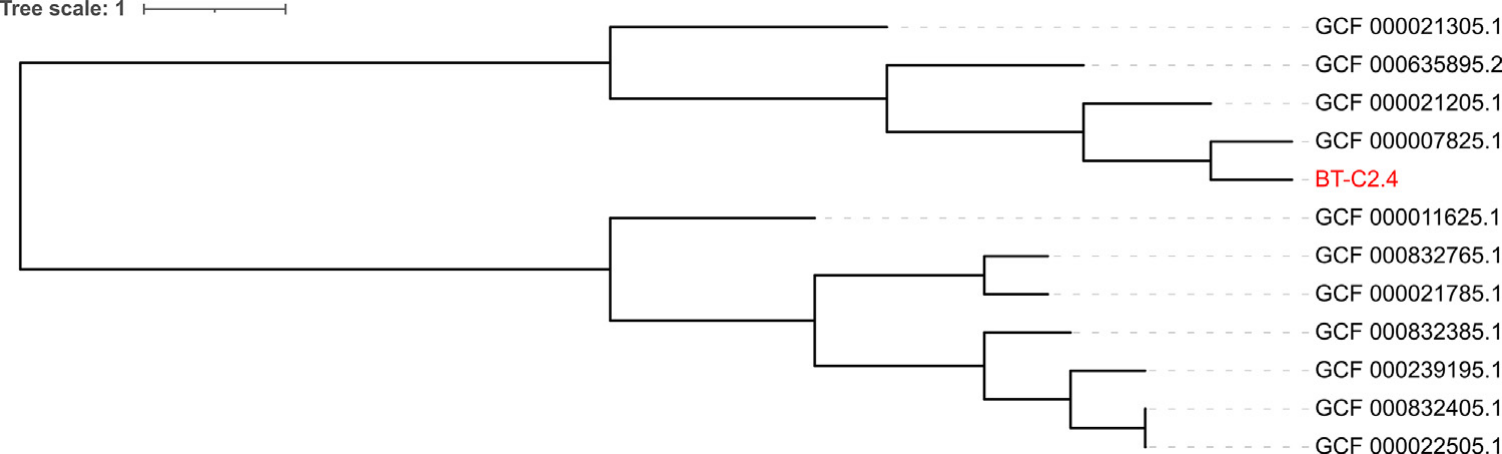
ANI-based phylogenetic tree showing the relationship between *B. cereus* BT-C2.4 and representative genomes of the *B. cereus* species complex.

Phylogenetic relationships based on average nucleotide identity (ANI) are presented in Fig. 3, showing the clustering of *B. cereus* BT-C2.4 with representative genomes of the *B. cereus* species complex.

**Fig. 4:**
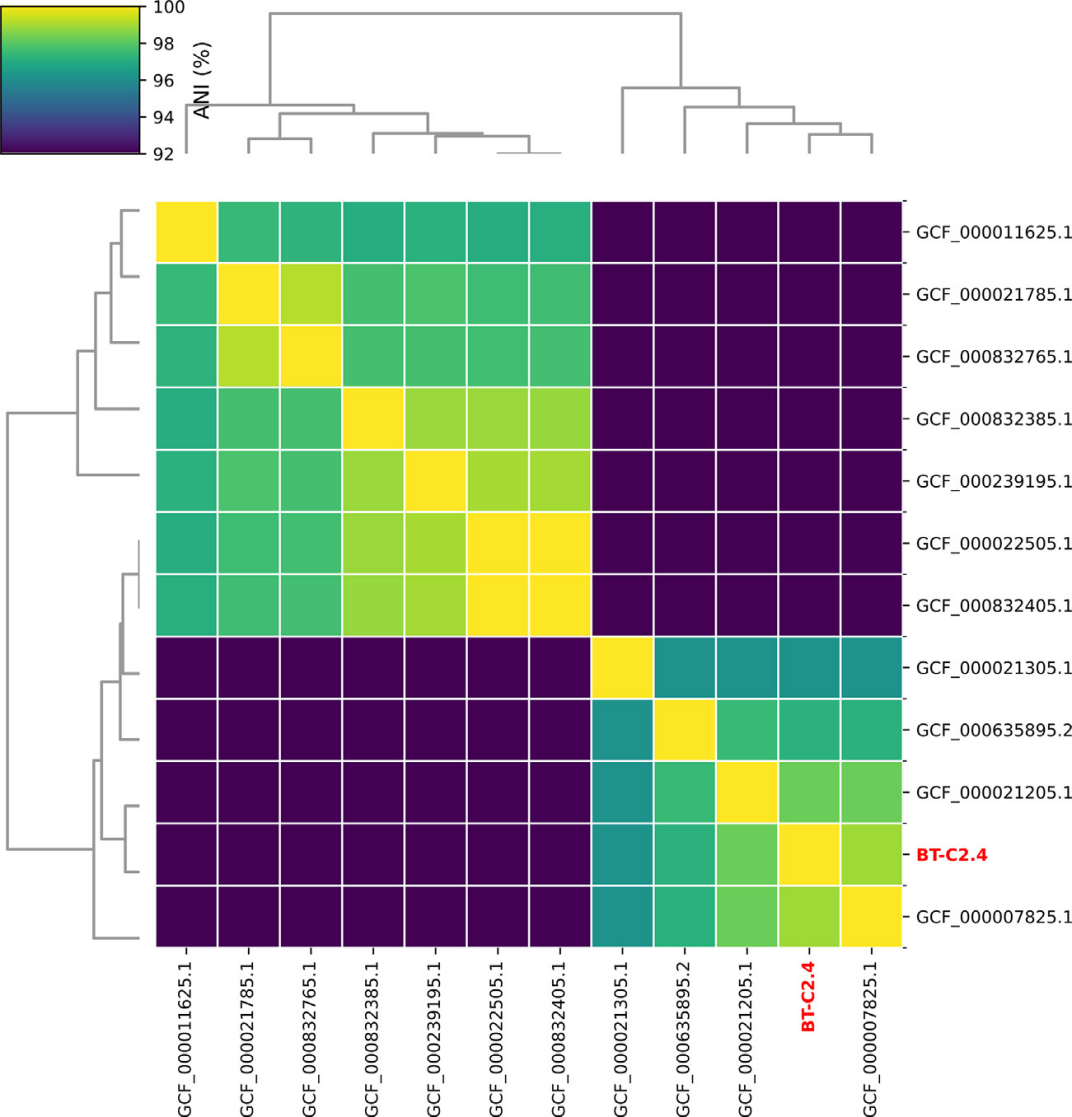
Average nucleotide identity (ANI) heatmap showing genomic similarity between *B. cereus* BT-C2.4 and closely related reference genomes.

The ANI heatmap further illustrates genomic similarity among the analyzed strains (Fig. 4).

The dataset also includes antimicrobial resistance gene annotation. Identified genes such as *BcI, BcII, fosB*, and *satA* are listed in Table 2.

**Table 2:** Antibiotic resistance genes detected in *B. cereus* BT-C2.4.

Gene	Function	Contig	Start	End
BcI	Class A β-lactamase	contig 1	178,901	179,842
BcII	Metallo-β-lactamase	contig 6	48,190	48,963
fosB	Fosfomycin resistance protein	contig 4	128,777	129,193
satA	Streptothricin acetyltransferase	contig 1	226,045	226,602

Virulence-associated genes detected in the genome, including *hbl, nhe, cytK2, plcA, sph*, and *entFM*, are summarized in Table 3.

**Table 3:** Virulence genes identified in *B. cereus* BT-C2.4.

Gene	Function	Contig	Start	End
hblA	Hemolysin BL binding component B	contig 1	286,834	288,234
hblB	Hemolysin BL binding component	contig 1	285,331	286,458
hblC	Hemolysin BL lytic component L2	contig 1	282,693	284,012
hblD	Hemolysin BL lytic component L1	contig 1	284,074	285,294
nheA	Non-hemolytic enterotoxin subunit A	contig 3	18,332	19,492
nheB	Non-hemolytic enterotoxin subunit B	contig 3	17,086	18,294
nheC	Non-hemolytic enterotoxin subunit C	contig 3	15,899	16,978
cytK2	Cytotoxin K2	contig 7	232,712	233,722
entFM	Enterotoxin FM	contig 4	54,243	55,523
sph	Sphingomyelinase C	contig 10	47,833	48,834
plcA	Phospholipase-related protein	contig 10	48,911	49,762

**Fig. 5:**
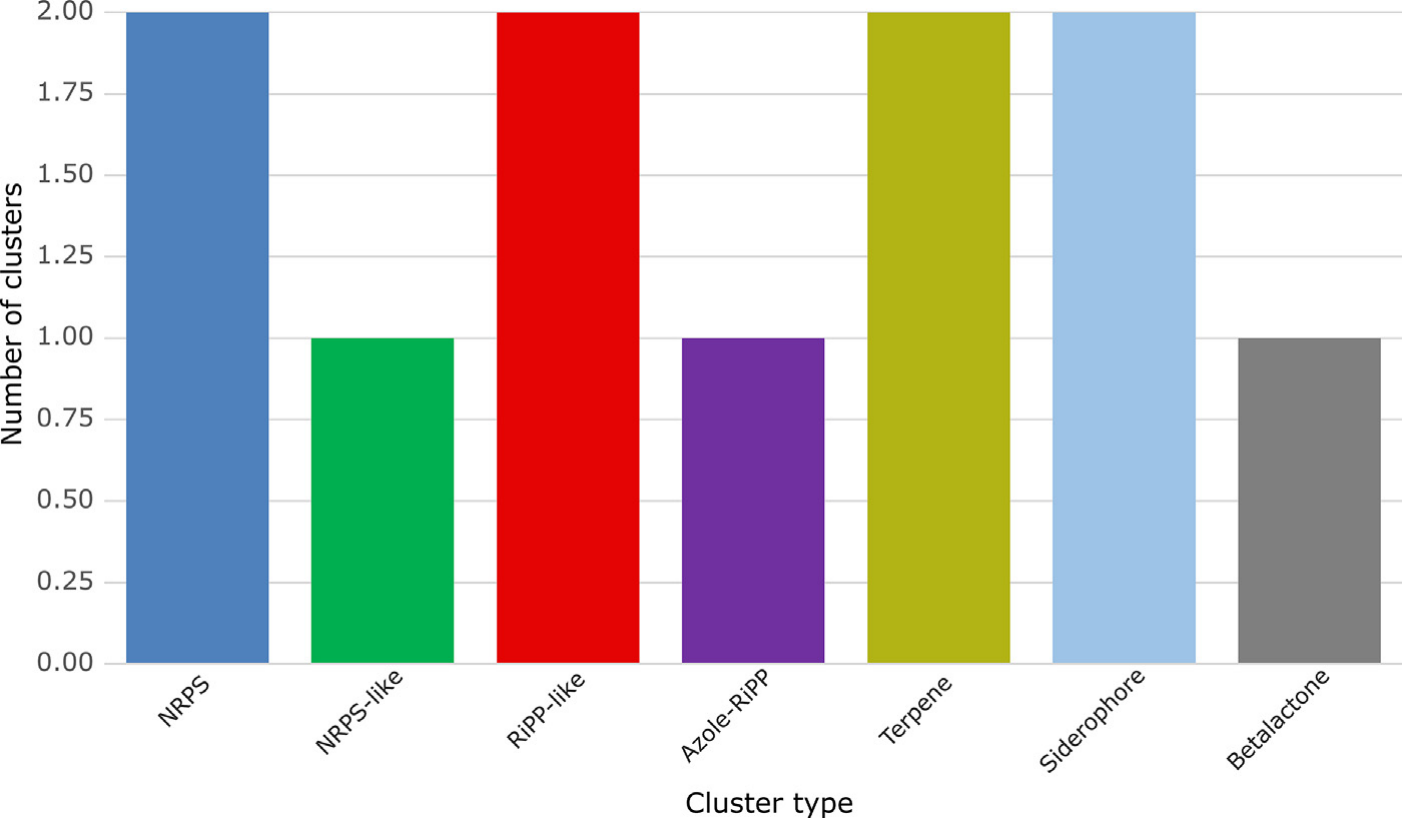
Distribution of predicted biosynthetic gene clusters in the draft genome of *B. cereus* BT-C2.4.

The dataset further contains predicted biosynthetic gene clusters. The distribution of these clusters, including siderophore-associated clusters such as petrobactin and bacillibactin, is shown in Fig. 5.

## 4 Experimental Design, Materials and Methods

### 4.1 Sampling and strain selection

The isolate *B. cereus* BT-C2.4 was recovered from aquaculture water samples using membrane filtration (0.45 µm), followed by cultivation on nutrient agar supplemented with cefotaxime (1 mg/L) at 37 °C for 24 h [1]. Extended-spectrum β-lactamase (ESBL) production was evaluated using the combined disk method following CLSI guidelines. Antimicrobial susceptibility testing and minimum inhibitory concentration (MIC) determination for amoxicillin were performed using the broth microdilution method, revealing a high resistance level (MIC = 2048 µg/mL) [2]. Interpretation of susceptibility results was conducted according to CLSI M100, 33rd edition (2023) [3]. The presence of ESBL associated genes was further screened by multiplex PCR [4]. Nutrient agar, LB agar, LB broth, tryptic soy agar, and brain heart infusion media were purchased from Merck (Darmstadt, Germany). Mueller–Hinton Broth 2 was obtained from Sigma-Aldrich (St. Louis, MO, USA). Cefotaxime used for bacterial isolation was purchased from Duchefa Biochemie (Haarlem, The Netherlands). Amoxicillin trihydrate used for susceptibility testing and degradation experiments was obtained from Bio Basic Inc. (Markham, Canada).

### 4.2 Species identification

Preliminary identification of the isolate was performed using MALDI-TOF MS (Bruker Daltonics, Germany), which served as a rapid screening method for species-level assignment prior to genome sequencing. Species identity was subsequently confirmed by whole-genome sequencing analysis, providing higher taxonomic resolution and enabling downstream genomic characterization. Taxonomic classification was initially performed using Kraken2 [5] and subsequently validated by average nucleotide identity (ANI) analysis using FastANI v1.34 [6], showing >98% similarity to *B. cereus* reference genomes.

### 4.3 Genome sequencing, assembly, and annotation

Genomic DNA was extracted using the DNeasy Blood & Tissue Kit (Qiagen, Germany). Whole-genome sequencing was carried out on the Illumina MiniSeq platform using 2 × 150 bp paired end reads, generating an average genome coverage of approximately 85×. Raw sequencing reads were quality checked using FastQC v0.12.1 [7] and processed using SeqKit v2.8.2 [8]. Low-quality reads and adapter-related artifacts were assessed during quality control prior to de novo assembly. The processed reads were subsequently used for genome reconstruction and downstream analyses. De novo genome assembly was performed using Unicycler v0.5.1 [9], which incorporates SPAdes for assembly construction. Assembly quality was evaluated using QUAST v5.2.0 [10], and genome completeness was assessed using BUSCO v5.7.1 [11]. Genome annotation was conducted to identify coding sequences and structural RNA genes, and the resulting annotations were used for subsequent analyses of antimicrobial resistance genes, mobile genetic elements, and biosynthetic gene clusters. Initial genome annotation was performed using Bakta v1.9.4 [12] prior to data submission. Following submission to NCBI, genome annotation was performed using the NCBI Prokaryotic Genome Annotation Pipeline (PGAP), and the annotation results from PGAP were used for reporting genome features in this study.

Antimicrobial resistance genes were identified using AMRFinderPlus v3.12.8 [13]. Mobile genetic elements were predicted using ISEScan [14], mobileOG-db [15], and geNomad [16]. Default parameters were used for all software unless otherwise specified.

## Limitations

The dataset is based on a single *B. cereus* isolate obtained from a specific aquaculture environment, which may limit its representativeness across different geographical locations. Genome assembly is draft quality and consists of multiple contigs, which may affect the resolution of genomic structural analysis. In addition, experimental data are limited to amoxicillin degradation under controlled laboratory conditions. Bacterial growth kinetics were not monitored during the degradation experiments; therefore, the relationship between cell growth and amoxicillin removal could not be evaluated from the current dataset.

## Ethics Statement

This study does not involve human subjects, animal experiments, or data collected from social media platforms.

## CRediT authorship contribution statement

**Hong Lat Luu:** Conceptualization, Methodology, Investigation, Data curation, Formal analysis, Writing – original draft. **Nhu Nguyet Phan:** Investigation, Validation, Data curation, Writing – review & editing. **Thanh Nhan Lam:** Formal analysis, Data curation, Visualization. **Trang Thi Phuong Phan:** Supervision, Project administration, Conceptualization, Writing – review & editing.

## Data Availability

Mendeley DataDataset for genome sequence and amoxicillin degradation of Bacillus cereus BT-C2.4 (Original data)
